# Dysregulation of MicroRNAs in Colonic Field Carcinogenesis: Implications for Screening

**DOI:** 10.1371/journal.pone.0045591

**Published:** 2012-09-25

**Authors:** Dhananjay P. Kunte, Mart DelaCruz, Ramesh K. Wali, Ashwaty Menon, Hongyan Du, Yolanda Stypula, Amir Patel, Vadim Backman, Hemant K. Roy

**Affiliations:** 1 Department of Medicine, NorthShore University HealthSystem, Evanston, Illinois, United States of America; 2 Department of Biomedical Engineering, Northwestern University, Evanston, Illinois, United States of America; Pontificia Universidad Catolica de Chile, Chile

## Abstract

Colorectal cancer (CRC) screening tests often have a trade-off between efficacy and patient acceptability/cost. Fecal tests (occult blood, methylation) engender excellent patient compliance but lack requisite performance underscoring the need for better population screening tests. We assessed the utility of microRNAs (miRNAs) as markers of field carcinogenesis and their potential role for CRC screening using the azoxymethane (AOM)-treated rat model. We found that 63 miRNAs were upregulated and miR-122, miR-296-5p and miR-503# were downregulated in the uninvolved colonic mucosa of AOM rats. We monitored the expression of selected miRNAs in colonic biopsies of AOM rats at 16 weeks and correlated it with tumor development. We noted that the tumor bearing rats had significantly greater miRNA modulation compared to those without tumors. The miRNAs showed good diagnostic performance with an area under the receiver operator curve (AUROC) of >0.7. We also noted that the miRNA induction in the colonic mucosa was mirrorred in the mucus layer fecal colonocytes isolated from AOM rat stool and the degree of miRNA induction was greater in the tumor bearing rats compared to those without tumors. Lastly, we also noted significant miRNA modulation in the Pirc rats- the genetic model of colon carcinogenesis, both in the uninvolved colonic mucosa and the fecal colonocytes. We thus demonstrate that miRNAs are excellent markers of field carcinogenesis and could accurately predict future neoplasia. Based on our results, we propose an accurate, inexpensive, non-invasive miRNA test for CRC risk stratification based on rectal brushings or from abraded fecal colonocytes.

## Introduction

Colorectal cancers evolve through a defined sequence of cellular and morphological events (hyperproliferative, nondysplastic epithelium→adenoma→carcinoma) which is choreographed by dysregulation of ∼15 critical molecular pathways [1]. These alterations occur through both endogenous (genetic, secondary bile salts, etc.) and exogenous (diet, smoking) insults which are diffuse (impacting the whole colon) leading to the well established concept of field carcinogenesis (aka field of injury, field effect, field defect). In this paradigm, while the fertile mutational field throughout the colon, the focal neoplastic lesions results from the stochastic occurrence of a critical molecular event (i.e. truncation of the adenomatous polyposis coli tumor suppressor gene) [2], [3].

Being able to detect field carcinogenesis is of major clinical and biological significance. Clinically, field carcinogenesis is used as a modality for risk stratification using a variety of biomarkers at the macroscopic (adenomas, aberrant crypt foci), microscopic (apoptosis/proliferation) and histologically normal (gene expression, proteomics etc) level. Biologically, this provides critical insights into early events in carcinogenesis especially in epigenetic silencing of tumor suppressor genes. One of the best established mechanisms of epigenetically silencing tumor suppressor genes in colorectal carcinogenesis is CpG island promoter hypermethylation [4], [5], [6], [7]. This has made clinical translation with implementation in fecal assays as a tool for colorectal cancer screening [8]. However, the accuracy has been suboptimal.

Recently, attention has focused on microRNAs (miRNAs) as important epigenetic modulators of gene expression during carcinogenesis. MicroRNAs are small non-coding, 18–25 nucleotides long RNAs that down-regulate gene expression through binding to the 3′ UTR and either degrading the mRNA or inhibiting the mRNA translation [9]. There has been an increasing interest in miRNAs in the pathogenesis of cancers. Dysregulation of >700 miRNAs has been implicated in carcinogenesis generally via epigenetic silencing of tumor suppressor genes or activation of proto-oncogenes (onco-miRs) [10]. Furthermore, the role of miRNAs in carcinogenesis has been bolstered by the observation that ∼50% of miRNAs are located in fragile areas (deletion/amplifications) [11]. There have been numerous studies showing that miRNAs are differentially expressed in colonic tumors. In CRC, miRNAs modulate many critical genetic pathways (e.g. EGFR AKT, PI-3 kinase, p53, IGF-1, COX-2, epithelial-mesenchymal transition, angiogenesis) [3]. The role of microRNAs in invasive CRC is underscored by the demonstration that tissue/serum microRNAs have prognostic value [12], [13], [14], [15].

Thus, while it is clear that miRNAs are important in colon carcinogenesis, the majority of the attention has focused on progression. The role of microRNAs in the early CRC phase has been relatively unexplored. While there are recent reports of microRNAs modulating the key “gatekeepers” of tumorigenesis (adenomatous polyposis coli or hMLH1 tumor suppressor) [10], [16], the role in field carcinogenesis (prior to tumorigenesis) has been largely unexplored.

In the present study, we utilize both a carcinogen (azoxymethane-treated rat) and genetic (polyposis in rat colon) models of colon carcinogenesis in order to rigorously evaluate the temporal alterations in mucosal microRNA expression. We noticed that microRNAs were profoundly altered at the premalignant stages of colon carcinogenesis and most remarkably these predicted future risk of neoplasia suggesting potential clinical implication. We underscored potential avenue of clinical translatability is a fecal assay and we demonstrate that miRNA modulation in mucus layer fecal colonocytes.

## Results

### MicroRNA Modulation in Uninvolved Mucosa of AOM Rats

**Figure 1:**
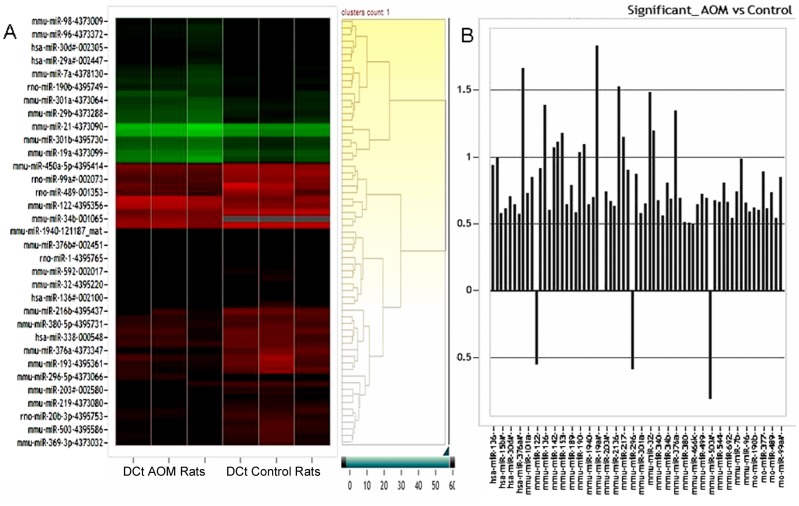
A: Hierarchical clustering of DCt values of differentially regulated miRNAs in the uninvolved colonic mucosa of AOM rat. **B:** Differentially expressed miRNAs in the AOM rat uninvolved colonic mucosa. Fold change was calculated as Log10 of Relative Quantitation.

We used the AOM-treated rat model given that the temporal nature is well defined. Specifically, it reliably requires ∼20 and ∼35–40 week post-carcinogen treatment to develop adenomas and carcinomas, respectively. To recapitulate the changes in field carcinogenesis in individuals at risk for concurrent neoplasia, we performed miRNA profiling of the uninvolved colonic mucosa (tumor field) of rats that were 40 weeks post-AOM injection. This demonstrated 63 microRNAs were significantly upregulated and 3 significantly downregulated (miR-122, miR-296-5p and miR-503) (Fig. 1A & 1B). The degree of miRNA modulation (Fold change) ranged from −0.7 to 1.7. The fold change was calculated as log10 RQ where RQ is 2^−ΔΔCT^.

### Confirmation of Modulation of a miRNA Panel in AOM Rats Uninvolved Colonic Mucosa

We observed significant miRNA modulation in the uninvolved colonic AOM rat mucosa using TLDA miRNA arrays. To corroborate these findings, we selected a panel of 12 most differentially expressed miRNAs and performed individual Taqman miRNA assays (Applied Biosystems). At the 40 week stage, all the 12 miRNAs tested manifested a statistically significant induction (Table 1). Among the miRNAs, miR-34a, miR-32, miR-376a, miR-384-3p, miR-29b and miR-142-3p were the highly overexpressed, with fold induction of 11.8, 24.2, 12.7, 14.4, 11.6 and 19.1, respectively.

**Table 1:** Confirmation of selected miRNA panel in AOM rat uninvolved colonic mucosa at neoplastic (40wk) stage.

miRNA	Fold change	p-value
miR-34a	11.8	0.00001
miR-21	7.6	0.00001
miR-18a	3.7	0.00159
miR-376a	12.7	0.00000
miR-19a	6.8	0.00003
miR-32	24.2	0.00000
miR-96	4.5	0.00002
miR-7b	7.4	0.00015
miR-384-3p	14.4	0.00434
miR-9	4.8	0.00046
miR-29b	11.6	0.00000
miR-142-3p	19.1	0.00001

### MicroRNA Expression can Predict Future Neoplasia

**Figure 2:**
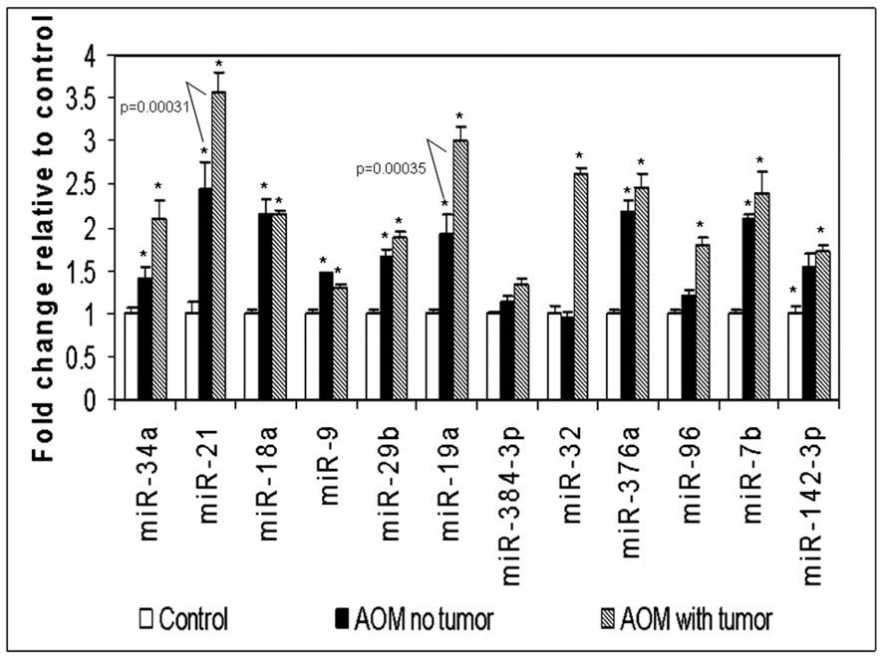
MicroRNA modulation in premalignant 16 wk miRNA predicts future neoplasia development. MicroRNA analysis was done on colonic biopsies from AOM rats at a preneoplastic (16 wk) time point. The rats were followed for 40 weeks and separated as tumor bearing and non-bearing AOM rats. The miRNA expression at the premalignant stage (16 wk) was then compared between the control rats and the AOM rats which eventually developed tumors or remained non-tumor bearing. *: p<0.05 when compared to normal saline controls. Specific p values are given in which AOM rats without tumors are compared to AOM rats with tumors.

The above data suggests that microRNA dysregulation is a marker of field carcinogenesis with regards to concurrent lesions. Clinically, field carcinogenesis can also be utilized to predict future neoplasia (metachronous lesions) [17]. By and large, the AOM rat model that only ∼50% of AOM-treated rats develop tumors. In order to investigate whether microRNAs could predict long-term risk (future neoplasia); we obtained colonoscopic biopsies at a pre-adenomatous time point (16 weeks post-carcinogen) and correlated with the miRNA expression at a time point when malignancy may occur in the same animals (40 weeks). We assayed the 12 miRNAs identified above for these longitudinal studies. In the 16 week colonic biopsies, we observed that while all miRNAs trended to increase (versus age-matched saline treated animals) although only 7 miRNAs (miR-34a, miR-21, miR-18, miR-376a, miR-19a, miR-9 and miR-29b) achieved statistical significance (fold inductions of 1.73, 2.72, 2.15, 2.26, 2.18, 1.53, and 1.71,respectively) (Table 2). All these microRNAs appeared to progressively increase from 16→40 weeks. Importantly, when the rats were stratified by their tumor bearing status (present/absent), the tumor bearing rats showed greater miRNA induction and miR-34a, miR-21, miR-19a, miR-32 showed a statistically significant effect compared to the saline controls (Fig. 2). We also compared the miRNA differences in the AOM rats which developed tumors with the AOM rats with no tumors. Out of the 12 miRNAs tested, miR-21 and miR-19a showed statistically significant differences (2.19 and 2.79 fold induction respectively) in the tumor bearing and non-bearing AOM rats. This data suggests that a small panel of miRNAs may have the potential of not simply identifying field carcinogenesis but also predict the future occurrence of neoplastic biomarkers of colon carcinogenesis and could predict the future neoplasia development.

**Table 2:** MicroRNA modulation in colonic biopsies of AOM rats at a premalignant (16 wk) stage.

miRNA	Fold change	p-value
miR-34a	1.7	0.011207
miR-21	2.7	0.00006
miR-18a	2.2	0.00034
miR-376a	2.3	0.00306
miR-19a	2.2	0.00028
miR-32	1.3	N.S.
miR-96	1.4	N.S.
miR-7b	0.8	N.S.
miR-384-3p	1.2	N.S.
miR-9	1.5	0.04500
miR-29b	1.7	0.015
miR-142-3p	1.3	0.042

N.S.: Non-Significant, N/A: Non-Applicable.

### MicroRNA Dysregulation in Mucus Layer Fecal Colonocytes

**Figure 3:**
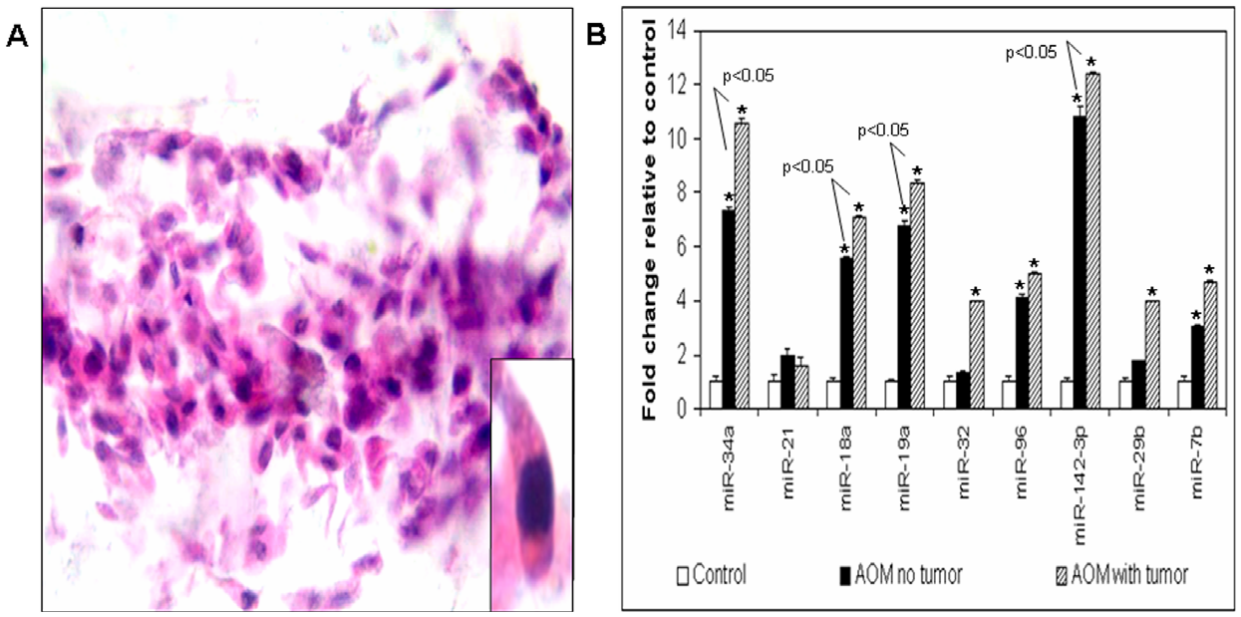
A: Hematoxylin and Eosin (H&E) staining of the fecal colonocytes isolated from rat stool. **B:** Differentially expressed miRNAs in the fecal colonocytes obtained from tumor bearing and tumor non-bearing AOM rats. *: p<0.05. Specific p values are given in which AOM rats without tumors are compared to AOM rats with tumors.

The earlier data indicated that miRNAs are good markers of field carcinogenesis and may be useful in predicting future neoplasia. In order to explore modalities of clinical implementation, we wanted to develop stool assay. We wanted to target mucus layer fecal colonocytes since these are presumed to be obtained from abrasion of normal epithelium (non-apoptotic) from the passage of formed stool bolus. We modified the technique from White and colleagues [18] and obtained morphologically normal looking colonocytes after hematoxylin and eosin (H&E) staining (Fig. 3A.). We utilized 40 week animals with three categories (saline-treated controls, AOM no tumors and AOM with tumors) based on rodent colonoscopy. We then obtained mucus layer fecal colonocytes 2–3 days post colonoscopy and quantified the expression of the 12-miRNA panel using individual Taqman assays. We noted that miRNAs miR-34a, miR-18a, miR-19a, miR-32, miR-96, miR-142-3p miR-29b and miR-7b were significantly upregulated in the AOM rat fecal colonocytes compared to those obtained from the saline controls and the degree of induction was greater in the tumor bearing AOM rats compared to the tumor non-bearing AOM rats (Fig. 3B). Furthermore, fecal colonocytes from the tumor bearing AOM rats showed a significantly higher induction of miR-34a, miR-18a, miR-19a and miR-142-3p (3.0, 2.3, 2.2 and 2.1 fold induction respectively) (p<0.05), Thus, it is evident that the miRNA dysregulation in the histologically normal colonic mucosa of the AOM rats was mirrored in the fecal colonocytes and the miRNA modulation was augmented by the presence of neoplasia in the colon thus supporting potential role as a minimally intrusive modality for field carcinogenesis detection.

### Predictive Ability of miRNA Markers

In order to identify a panel of miRNAs which accurately discriminates controls from AOM rats, we performed univariate logistic regression analysis of the miRNA DCt values from control and AOM rat colonic biopsies obtained at a premalignant time point (16 weeks post-AOM). We also performed receiver operator characteristic curve analysis (ROC) of the normalized cycle threshold (Ct) values of the miRNAs using STATA software program. Out of the 12 miRNAs tested, four (miR-21, miR-18a, miR-29b, and miR-19a) were significantly different between AOM and Saline group (p<0.02). According to Hosmer & Lemshow test, an AUC>0.7 is regarded very good discrimination, >0.8 excellent discrimination, and >0.9 distinguished discrimination. Based on these criterion miR-21 achieved distinguished predictive ability with a 0.914 AUC, miR-18a and miR-19a achieved excellent predictive ability with a 0.877 and 0.872 AUC respectively, miR-29b achieved an almost excellent predictive ability with a 0.789 AUC. We further attempted LASSO (least absolute shrinkage selection operator) approach among the ten markers and found three microRNAs (miR-21, miR-18a, and miR-19a) as the most sensitive. LASSO was used here to avoid over-fitting problem, given that we had relatively small sample size with large number of candidate markers. The internal validity of the regression model with the three markers was assessed by using bootstrapping techniques. Random bootstrap samples were drawn with replacement from the full sample (150 replications). This validation replicates the situation in which the prediction model based on our rats is applied to a group of similar rats. The area under the ROC curve was 0.914 on the full data set and 0.870 after this procedure.

Similarly, in the fecal colonocytes, the miRNAs miR-34a, miR-18a and miR-19a, showed an AUROC of 0.926, 0.918 and 0.968, respectively. While clearly preliminary, this data supports the promise of using a panel of miRNAs for field carcinogenesis detection.

### MicroRNA Profiling in Pirc Rats

**Figure 4:**
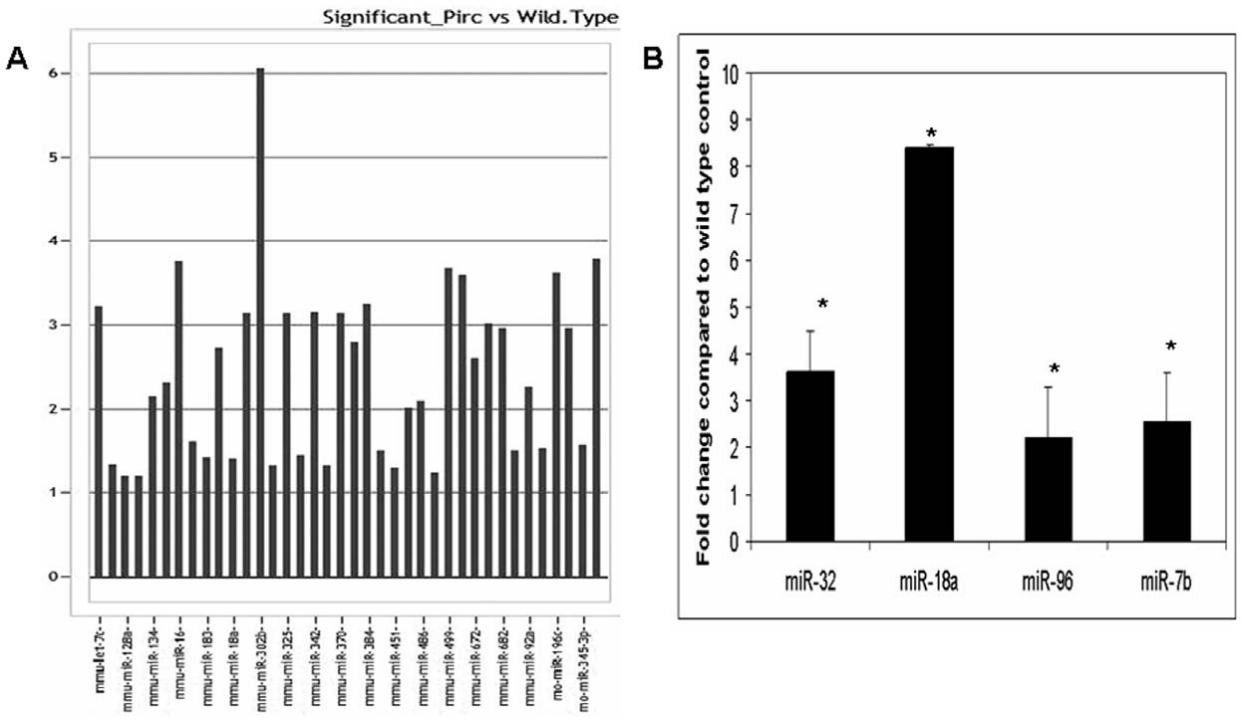
A: Significantly modulated miRNAs in the Pirc rat uninvolved colonic mucosa. Fold change was calculated as Log10 of Relative Quantitation. **B:** Differentially expressed miRNAs in the fecal colonocytes obtained from Pirc rats. *: p<0.05.

We had earlier noted that miRNAs were dysregulated in the chemical carcinogen-induced (AOM) model of colon carcinogenesis and serve as excellent markers of field carcinogenesis. We wanted to see if this is mirrored in the genetic model of colon carcinogenesis. Recently, the Polyposis in the Rat Colon (Pirc) model is reported as an excellent genetic model of CRC which contains a germline truncation in the adenomatous polyposis coli (APC) tumor suppressor gene, the initiating event in most colorectal cancer. Furthermore, just as in humans analogue (familial adenomatous polyposis), all affected individuals develop predominantly colonic neoplasia. We did miRNA profiling of the Pirc rats from the colonic biopsies obtained at a time point prior to adenoma determination (12 wks with confirmatory negative colonoscopy). We noted a robust miRNA modulation in the Pirc rats wherein 38 miRNAs were significantly induced (Fig. 4A). We noted that the miRNA modulation obtained in Pirc rats was more dramatic than in AOM rat model which might reflect the higher penetrance of this germline mutation versus carcinogen treatment. Intriguingly, while the AOM and Pirc models shared some miRNAs that were dysregulated, others were distinct suggesting that this may be due to differences related to genetic heterogeneity in tumorigenesis. Thus, the miRNA modulation in the CRC field effect occurs regardless of the cancer-initiation model.

### MicroRNA Modulation in the Pirc Rat Fecal Colonocytes

As a proof of concept, we also determined whether the in the Pirc rat miRNA modulation observed in the colonic mucosa is mirrored in the normally defecated fecal colonocytes. We selected 6 miRNAs overexpressed during field carcinogenesis and longitudinally evaluated these markers in the mucus layer fecal colonocytes. Four of these miRNAs were observed to be significantly induced in the Pirc rat fecal colonocytes when compared to stool from age matched wild type rat (Fig. 4B), thus mirroring the results obtained in the colonic mucosa.

## Discussion

We demonstrate herein that a number of microRNAs were dysregulated in the predysplastic (microscopically normal) colonic mucosa. Our approach was relatively comprehensive utilizing the TLDA array platform and validated by individual miRNA Taqman assays. Furthermore, this was not model specific since miRNA dysregulation was also noted both in the well-validated AOM-treated rat model and the genetic Pirc model. The striking finding was that not only were these micro RNAs dysregulated in cancer, but also at the premalignant time point and predict which AOM-treated animals would actually develop tumors in the future making this potentially relevant to colorectal cancer screening (identifying at risk patients to interrupt the adenoma→carcinoma progression). Finally, with regards to clinical implementation perspective, we demonstrated that this could be conducted from mucus layer fecal colonocytes representing a non-invasive method of detecting field carcinogenesis.

There has been considerable interest in the role of microRNAs in colon carcinogenesis, although most studies have focused on progression and therapeutics. For instance, there is clear evidence that dysregulation of a variety of miRNAs may result in progression as many miRNAs are progressively altered between adenomas and carcinomas [19], [20]. Large number of studies has shown that miRNAs play an important prognostic role in cancer. For instance, Schetter and colleagues showed that miR-21 corresponded to colorectal cancer related mortality [15]. Furthermore, Nielsen and colleagues recently demonstrated that stromal microRNA expression was able to prognosticate the clinically vexing issue of stage 2 disease [21]. Finally, from a therapeutic perspective, recent reports have suggested that resistance to one of the stalwart drugs against colorectal cancer is driven by mir-21 and manipulation of miRNAs can overcome resistance [22].

Emerging evidence suggests that microRNAs may be modulated early in colon carcinogenesis. For initiation of colon carcinogenesis, the typical “gatekeeper” genetic events are either APC or less frequently mismatch repair genes (generally hMLH1) [23]. While these are typically inactivated mutationally or through methylation, there is some suggestion that miRNA epigenetic silencing may also have a role. For instance, recent reports have indicated that both APC and MMR gene expression is regulated by miRs (e.g. miR-135 and miR-155, respectively) [10], [16]. Moreover, SND1, a component of RNA-induced silencing complex, has been noted to be upregulated in the putative earliest morphological of colon carcinogenesis, the aberrant crypt foci [24].

Given the emerging data that microRNA dysregulation may play an integral role in early neoplastic transformation, the question that follows is whether microRNA could be altered in field carcinogenesis. It is apropos to note that microarray studies have shown that critical mediators of neoplastic transformation including COX-2, osteopontin etc. are dysregulated in the microscopically normal mucosa (i.e. field effect) [25]. Additionally, more recent studies have suggested that loss of DNA mismatch repair proteins (hMLH1 and MSH 2) occurs in the microscopically normal mucosa in patients harboring neoplasia elsewhere in the colon [10]. This is particularly intriguing because, as previously discussed, microRNAs have been shown to be potential mediators of these proteins. Consonant with the role of microRNA, there have been some early reports that the majority of colorectal cancer patients had epigenetic silencing of the intronic micro RNA miR-342 in the uninvolved mucosa (with >80% of tumor tissue manifesting concomitant loss) [19]. Similarly, Balaguer and colleagues noted that miR-137 was methylated in 81% of CRCs and while much less frequently seen in the histologically normal mucosa was ∼3 fold higher than in corresponding mucosa that was neoplasia-free [26]. Moreover, microRNAs in the uninvolved mucosa can be modulated by chemopreventive agents [27]. Our study expands this work by using microRNAs in animals with neoplasia. Furthermore, our data is the first to focus on the performance characteristics in discriminating between neoplasia-free versus those with field carcinogenesis signatures.

The potential clinical implication is to develop a minimally intrusive solution to the critical issue of colorectal cancer risk analysis. For instance, techniques such as colonoscopy has been quite effective but the group for which it is recommended is quite expansive (the entire population over age 50). Unfortunately, the prevalence of screen relevant neoplasia (advanced adenoma or early stage cancer) is low (∼5–6%) and thus the vast majority of the expensive, invasive and potentially morbid colonoscopies are unproductive from a cancer death prevention perspective. The inefficiency of resource utilization (funds, endoscopic capacity) is juxtaposed with the observation that most of the population does not undergo colonoscopy. This may be a key factor in why CRC remains the second leading cause of cancer deaths among Americans and has underscored the urgency of finding a minimally intrusive (fecal) pre-screen for colonoscopy. For instance, fecal occult blood test identifies patients at higher risk and therefore necessitates colonoscopy but its diagnostic performance for advanced adenomas (defined as size ≥1 cm or >25% villous features or presence of high grade dysplasia) is only 11% [28], [29]. Recent advances such as immunohistochemical tests for hemoglobin, mutational (APC, K-ras, p53) or methylation (vimentin) analysis have represented modest steps forward but still considered suboptimal for CRC prevention efforts [30]. This underscores the interest in exploiting microRNAs, whose role in the carcinogenesis is well-established, as a screening tool.

We believe that our data represents a significant step forward in translating microRNAs to the screening arena. Specifically, the tissue microRNA (field carcinogenesis detection) showed promising diagnostic performance. One could consider clinical implementation with a simple digital rectal exam with colonocytes captured with a modified examination glove. Even less intrusive would be assay the stool as discussed above. MicroRNAs represent a promising target because these small RNAs are more resistant to degradation than mRNA. Previous studies have demonstrated feasibility of this approach. For instance, in a landmark study, Goel and colleagues [31] showed that microRNAs could be detected in the stool and this was recently replicated by Koga et al [32]. Our approach is a potentially important improvement in that instead of evaluating the small number of tumor products in the stool (the “needle in the haystack”); we target the process of field carcinogenesis which is ubiquitous among colonocytes in the fecal stream. We do this by using the isolated mucus layer fecal colonocytes [18] which has been posited to be acquired by abrasion of the normal colonic epithelium by the solid stool bolus. Furthermore, there is an augmentation of microRNA in tumors suggesting improved fecal diagnostics for concurrent lesions.

While large proportion of colon cancer incidence is sporadic, a small percentage (25%) of CRC cases arise through genetic/hereditary causes (Familial Adenomatous Polyposis (FAP) and hereditary non-polyposis colorectal cancer (HNPCC)). In the present study, we used two animal models of CRC to study the role of miRNAs in both sporadic (carcinogen-induced) and genetic causes of CRC. The Azoxymethane treated rat model is a well-validated model of carcinogen induced experimental colon carcinogenesis. Thus, this represents an excellent model to study the role of miRNAs in CRC field carcinogenesis at premalignant and malignant stages. In the AOM rat model only ∼ 50% of AOM-treated rats develop neoplasia by 40 weeks. This gave the opportunity to study the miRNA expression in tumor-bearing and tumor non-bearing rats, both in colonic mucosa as well as fecal colonocytes. Recently, a new genetic model of CRC was developed in rat (Pirc model) that provides an important tool for CRC research. The Pirc rats have mutated APC gene thus not just genetically recapitulating human disease such as familial adenomatous polyposis but also most sporadic colonic neoplasia. We compared the miRNA expression in both the AOM rat and the Pirc rat model and observed robust miRNA modulation in the uninvolved colonic tissues (CRC field). We also noted that these changes were mirrored in the fecal colonocytes miRNA assays, thus indicating that the fecal miRNAs may be good markers of CRC field carcinogenesis.

There are several limitations to this study that should be acknowledged. Firstly, this data was confined to rodent models and relevance to human neoplasia needs to be confirmed. On the other hand, the AOM-treated rat has been the stalwart preclinical studies in experimental colorectal carcinogenesis and the model specific issues are mitigated by corroboration with the genetic Pirc rat model. Secondly, while we noted similar miRNA modulation in AOM rat mucosa and fecal colonocytes, the sample size was modest. In order to establish the fecal miRNA analysis as the marker of field carcinogenesis and CRC risk prediction, larger studies would be needed. Thirdly, for the fecal microRNA studies, the contamination (especially at later time points) with established tumors is possible but lessened by the assessment with rat colonoscopy which visualized the majority of the bowel (except for cecum). Finally, while we present a compelling preliminary data on miRNA modulation in fecal colonocytes, for practical application of the fecal miRNA test, this should be tested in human patients with and without adenomas/carcinomas.

In conclusion, we present herein evidence that microRNAs are progressively altered during colon carcinogenesis and are dysregulated at a predysplastic time points in the AOM-treated rat model. We confirm this fact in the histologically normal mucosa in humans. Importantly, the diagnostic accuracy was strong for both synchronous and metachronous lesions (field carcinogenesis ± tumor elaborated miRNAs). Furthermore, we demonstrate proof of concept that this approach can be non-invasively utilized through targeting mucus layer fecal colonocytes. These studies represent an important first step in developing a novel risk stratification technique and may provide important insights into the biology of early colon carcinogenesis.

## Methods

### Animals

All animal studies were performed in accordance with the Institutional Animal Care and Use Committee (IACUC)of NorthShore University Health System. All the animal protocols involving AOM rats and the Pirc rats were approved by the IACUC. Fisher 344 rats (150–200 g) received 2 weekly intra-peritoneal injections of azoxymethane (AOM) (15 mg/kg; Midwest Research Institute, Kansas City, MO). The rats were euthanized at forty weeks. In a separate experiment, colonic biopsies were obtained from the AOM rats at a 16 wk time point and rats were followed for 40 wks and tumor counts were taken. Total RNA from the colonic mucosa and colonic biopsies was isolated using Ribopure RNA kit (Ambion) using manufacturer’s instructions. Wild type Fisher 344 and Pirc rats were obtained from Taconic Laboratories (Hudson, NY). They were maintained on AIN 76a diet (Harlan Teklad, Madison, WI). The wild type and Pirc rats were euthanized after 24 weeks. The CRC progression in AOM rats as well as Pirc rats was monitored by colonoscopy using ColoView colonoscope (Storz) and colonic biopsies were obtained at the indicated time points.

### MicroRNA Profiling in AOM Rats

The concentration and purity of total RNA was measured by spectrophotometry at OD 260/280. Total RNA from rat colonic mucosa and colonic biopsies was reverse transcribed by priming with Rodent MegaPlex RT Primers (Applied Biosystems, Foster City, CA) using Taqman miRNA reverse transcription kit (Applied Biosystems) according to the manufacturer’s instructions. The cDNA diluted in Universal PCR Master Mix II (Applied Biosystems) was then loaded on to the Taqman Low Density Array (TLDA) microfluidic cards (Applied Biosystems) and run on ABI 7900 HT real time PCR system using manufacturer’s instructions. Relative concentrations of miRNAs were calculated using the comparative (2^−ΔΔCt^) method. The fold change was calculated as log10 RQ where RQ is 2^−ΔΔCT^. Real time PCR data analysis was done using the RQ Manager 1.2.1 (Applied Biosystems) and RealTime StatMiner software (Integromics, Philadelphia, PA).

### Fecal Colonocyte Isolation

The fecal colonocytes were obtained by isolating the mucus layer on the outer surface of the normally defecated rat stool using a modified protocol described by White and colleagues [18]. Briefly, stool was suspended in PreservCyt solution (Hologic) and gently agitated to dislodge the mucus layer from the outer surface of the stool and the suspension was then centrifuged at 800 rpm for 5 min. at 4°C and the supernatant was discarded. The pellet was resuspended in PreservCyt solution (Hologic) (5 ml/g of stool) and incubated for 45 minutes at room temperature to preserve and fix the colonocytes. After incubation, the suspension was successively filtered through 300 µm polypropylene mesh (Fort Atkinson, WI) (to remove large debris) and 125 µm mesh (Small Parts, Inc (Seattle, WA) (to obtain the mucus layer). The mucus layer retained on the 125 µM filter mesh contained the embedded colonocytes. The cells were resuspended in TRI reagent and RNA isolation was carried out using Ribopure RNA kit (Ambion) according to the manufacturer’s instructions.

### Taqman miRNA Assays and Real Time RT-PCR

Expression of selected miRNAs was assessed in the colonic biopsies, uninvolved colonic mucosa and fecal colonocytes using individual Taqman miRNA assays (Applied Biosystems) run on the Corbett Rotor-Gene® 6000 real time PCR system (Qiagen) using miRNA reverse transcription kit and Universal PCR Master Mix (Applied Biosystems) following the manufacturer’s instructions. Fold change in miRNA expression was calculated by the 2^−ΔΔCT^ method.

### Statistical Analysis

Statistical significance for the individual miRNA expression was performed with Microsoft Excel and the area under the receiver operator curve (AUROC) was calculated using STATA 8 software. A two tailed Student’s t-test was utilized with p value of <0.05 was determined as significant.
